# Skin Microbiome Shifts in Various Dermatological Conditions

**DOI:** 10.3390/jcm14176137

**Published:** 2025-08-30

**Authors:** Conan H. Lee, Mildred Min, Sami S. Jin, Raja K. Sivamani

**Affiliations:** 1 School of Medicine, University of California Davis, 4610X St., Sacramento, CA 95817, USA; cohlee@health.ucdavis.edu; 2 College of Medicine, California Northstate University, 9700 W Taron Dr., Elk Grove, CA 95757, USA; mildred.min6124@cnsu.edu; 3 Integrative Skin Science and Research, 1495 River Park Drive, Sacramento, CA 95819, USA; 4 School of Medicine, University of California San Diego, La Jolla, CA 92093, USA; sej004@health.ucsd.edu; 5 Pacific Skin Institute, 1495 River Park Dr Suite 200, Sacramento, CA 95815, USA; 6 Department of Dermatology, University of California-Davis, 3301 C St #1400, Sacramento, CA 95816, USA

**Keywords:** skin microbiome, shift, microbiota

## Abstract

**Background/Objectives**: The human skin provides a protective barrier composed of bacteria, fungi, viruses, and archaea that prevents the invasion of harmful organisms. Recent advancements in genomic sequencing have allowed for greater accuracy of species detection. This review aims to summarize the most up-to-date skin microbiome shifts in various dermatological diseases. **Methods**: A systematic search was conducted to examine microbiome shifts comparing lesional and nonlesional or diseased and healthy skin across various dermatological conditions. A literature search was conducted on PubMed, Web of Science, and Embase Databases from inception through April 2024, yielding 38 studies. **Results**: Staphylococcus aureus was reported unanimously in all skin conditions. Most studies in this review, except those investigating acne vulgaris, showed a decreased microbiome diversity in diseased skin. Finally, there was a greater shift in the proportion of pro-inflammatory bacterial and fungal strains. **Conclusions**: The skin microbiome is significantly altered in the progression of numerous dermatological diseases. Diversity of the skin microbiome is decreased, and there is an increased proportion of pro-inflammatory bacterial and fungal strains. The skin microbiome also provides a more comprehensive understanding of the development and progression of many inflammatory skin diseases. Prebiotic treatments may propose a much safer and cheaper alternative to antibiotics, which can have highly toxic side effects and negative implications for public health regarding antibiotic resistance. More research is required to fully understand both the microbiome changes and efficacy and viability of using probiotic treatments to restore the skin microbiome, thereby improving patient outcomes in all dermatological conditions.

## 1. Introduction

The human skin provides an effective physical barrier that prevents the invasion of harmful organisms and foreign substances. The skin microbiome contains billions of microorganisms composed of bacteria, fungi, viruses, and archaea [1]. The skin microbiome consists mainly of four types of bacteria: Actinobacteria (52%), Firmicutes (24%), Proteobacteria (16%), and Bacteroidetes (6%) [2]. Representatives of Cutibacterium, Staphylococcus, and Corynebacterium genera constitute 45 to 80% of the skin microbiome [3]. These microbes produce antimicrobial peptides that help contribute to inflammatory homeostasis and regulation of immune responses, which are disrupted in diseases presenting with a dysbiosis of the skin microbiome such as acne, atopic dermatitis, psoriasis, and rosacea [4,5].

Currently, there are different methods in characterizing the skin microbiome. One method involves 16S ribosomal RNA (rRNA) sequencing, which uses PCR to amplify the 16S rRNA region with primers [6]. The accuracy of this method can be limited by horizontal gene transfer of various bacteria strains [7]. On the other hand, shotgun whole-genome sequencing (WGS) is a more expensive method that uses random primers to sequence overlapping regions of a genome [8]. A comparative study demonstrated that WGS was more effective than 16S rRNA sequencing, with an enhanced detection of the species, diversity, and prediction of genes [9]. Nonetheless, research surrounding the microbiome is continuously evolving and expanding, and with the advancements in sequencing techniques, there is a more comprehensive understanding of the role of the skin microbiome in dermatological conditions. With these advancements, there is more potential in utilizing the microbiome to treat dermatological diseases. It is also important to highlight the underappreciation of fungi research on inflammatory skin conditions. Expanding the research focus outside of just bacteria will allow us to gain a better understanding of antifungal immunity surrounding T cells and the pathogenesis of many skin conditions [10]. To our knowledge, the last scoping review observing the shift in skin microbiome across multiple dermatological diseases was published in 2018. This scoping review aims to summarize the most recent research to critically analyze and summarize the evidence discussing how the skin microbiome is shifted in various dermatological diseases.

## 2. Materials and Methods

**Figure 1:**
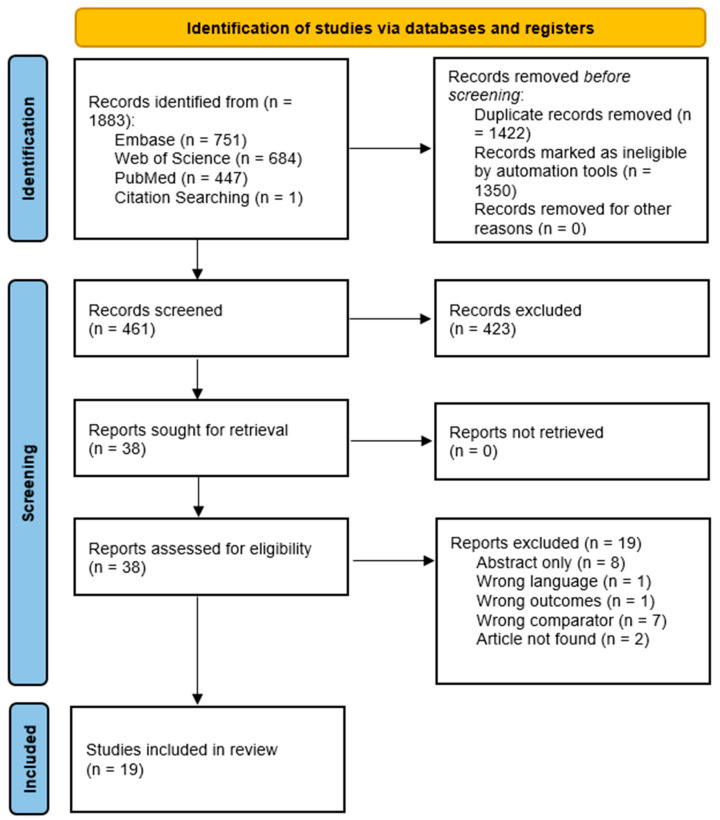
PRISMA-ScR flow diagram.

We adhered to the Preferred Reporting Items for Scoping reviews and Meta-analysis extension for Scoping Reviews (PRISMA-ScR) [11] and consulted with a trained librarian from the University of California, Davis School of Medicine. A systematic search was performed on PubMed/MEDLINE, Web of Science, and Embase with the following keywords: humans AND (microbiome OR microbiota) AND (atopic dermatitis OR psoriasis OR acne OR dermatitis OR rosacea OR hidradenitis suppurativa OR skin) AND (shift OR Change). Specific skin conditions were included in the systematic search based on their high prevalence and clinical impact. We applied database filters to include only records categorized as Book and Documents, Clinical Trials, Meta-Analyses, Randomized Controlled Trials, and Reviews. Eligible studies involved prospective and association-based studies comparing the microbiome analysis of healthy and diseased or lesional or nonlesional skin consisting of published manuscripts in English from database inception to April 2024. Animal studies, case studies, non-English literature, systematic reviews, and studies with absent or unclear findings of skin microbiome analysis were excluded. Two independent reviewers (C.H.L. and M.M.) completed the title and abstract screening using the COVIDENCE online scoping review software platform version 2.0 (Veritas Health Innovation, Melbourne, Australia). The reviewers were blinded by each other’s decisions and discrepancies were resolved through a consensus. Reviewers collected information including author (year), skin disease, sample size, measures (e.g., SCORAD, Shannon Diversity index, PD_whole_tree index), and findings of differences in microbiome profiles and its relative abundance. This review was not registered in PROSPERO or any other registry. This scoping review was conducted in accordance with the PRISMA-ScR guidelines. A completed PRISMA-ScR can be found in the Supplementary and the flow diagram is provided below (Figure 1).

## 3. Results

The search resulted in 1882 titles. One additional title was identified through citation searching and included for review. After removal of duplicate and ineligible studies, 461 studies were screened for inclusion: 423 studies were excluded due to irrelevancy, and the remaining 38 full-text studies were assessed for eligibility through our inclusion criteria. Nineteen studies were included in this review, and the study characteristics can be found in Table 1.

### 3.1. Dermatologic Conditions

#### 3.1.1. Acne Vulgaris

Acne vulgaris is the most prevalent chronic inflammatory disease involving *Cutibacterium acnes* (previously known as *Propionibacterium acnes*) colonization of the pilosebaceous follicle [31,32]. *C. acnes* plays a major role in the inflammatory pathogenesis of acne through recruitment of lymphocytes, neutrophils, and macrophages, all of which can further damage the follicular epithelium [33]. *Cutibacterium acnes* also secretes a lipase that metabolizes triglycerides into glycerol and fatty acids, forming comedones and inflammation of the skin [34].

From our scoping review, there are two studies on the microbiome shifts in acne. Both found an increased amount of *Staphylococcus* bacteria. A double-blind, split-face RCT investigated the microbiome shift in acne and compared it with unaffected skin [12]. This study had numerical data, showing a 33.87% composition of *Staphylococcus* in lesional skin compared to 26.85% in nonlesional skin. Additionally, *Firmicutes* increased with a 52.01% composition in lesional versus 47.01% in nonlesional skin. *Proteobacteria* was significantly decreased, with 28.90% in lesional skin compared to 34.10% in nonlesional skin. Similar Shannon diversity index scores were reported for both lesional and nonlesional skin.

A prospective pilot study looked at the microbiome of preadolescent acne, comparing diseased skin with healthy controls [13]. The alpha diversity index score was reported to be higher in the diseased state compared to the control in four sites (midline forehead, dorsum of the nose, medial left cheek, and the chin). The retroauricular crease was the only exception in which the alpha diversity score was not higher in the diseased state. Although no numerical data is reported, there was a reported increase in *Cutibacterium* (*Propionibacterium*) in diseased versus healthy skin.

#### 3.1.2. Atopic Dermatitis

Atopic dermatitis (AD) is a common inflammatory skin disorder with debilitating rash and pruritus [35]. Historically, microbes such as *S. aureus* and *Malassezia* are associated with the pathogenesis of AD [36,37]. Skin colonization by *S. aureus* has been associated with an increased production of IgE due to targeting of IgE molecules to *S. aureus* toxins [38,39].

A total of nine studies investigated the microbiome of AD on lesional versus nonlesional and diseased versus healthy skin. All studies agreed that there was a significant reduction in the Shannon diversity index of AD compared to nonlesional or healthy skin. One RCT specifically showed that lesional skin had an index value of 2.9 versus the healthy control, which had an index value of 4.49 [16]. This study also discovered that there was an increased amount of human beta defensin 2 (hBD-2) in lesional skin. A split-face RCT found a negative correlation between the Shannon diversity index and Severity Scoring of Atopic Dermatitis (SCORAD) in lesions of AD [21]. Four studies found an increased amount of *S. aureus* in lesional skin [14,15,18,19]. Of these four studies, a single-blinded RCT reported nonlesional skin with less than 25% composition of *Staphylococci*, whereas lesional skin had a composition of 60 to 70%. In another study, there was an increased baseline total bacteria density in diseased skin compared to healthy skin by approximately ten-fold [18]. In the third RCT, *S. aureus* comprised 72.5% of the whole species in lesional skin, which was significantly higher than in nonlesional skin (*p* = 0.0014) [19]. The final study demonstrated that the relative abundance of *S. aureus* in AD patients was significantly higher in lesional (36.35%) and nonlesional (7.20%) compared to healthy controls (2.08%, *p* < 0.001) [15]. In a non-randomized controlled trial investigating children with AD versus healthy children, CHROMagar analysis of *S. aureus* and *Malassezia* demonstrated increased *S. aureus* (11.0/25 cm^2^ versus 5.0/25 cm^2^ in healthy controls) and *Malassezia* species (1.5/25 cm^2^ versus 1.0/25 cm^2^ in healthy controls) in AD skin [20]. Furthermore, two studies correlated severity of AD to total number of *S. aureus* on lesional skin, demonstrating that the burden of *S. aureus* may be associated with disease burden [15,20]. Other reports include significantly decreased density in *Corynebacterium*, *Propionibacterium*, and *Lactobacillus* species [16,20,21]. Interestingly, one study focused on the mycobiome differences in lesional versus nonlesional skin in AD [17]. This study found four fungal genera that were present only in lesional skin and these included Alternaria, Coniosporium, Debaryomyces, and Capnodiales. Moreover, there were several bacteria that were significantly correlated with pathogenic fungal species in lesional skin. For example, *Corynebacterium kroppenstedtiian* and *Staphylococcus pettenkoferi* were positively correlated with *Candida* spp., and *Pseudomonas* spp. were correlated with *Aspergillus* and *Candida* spp. [17].

#### 3.1.3. Androgenetic Alopecia

Androgenetic alopecia (AGA) is a progressive hair loss disorder with an influence from predetermined genetics and an excessive response to androgens [40,41]. Recent research has found an increase in *C. acnes* and *Burkholderia* in AGA, and an increase in *P. acnes* may be associated with an elevated immune response and gene expression in the hair follicle [42]. Although increased *Malassezia* has been associated with disease progression of AGA, studies suggest that a host predisposition in addition to microbiome shift in *Malassezia* is required for development of AGA [43,44].

One non-randomized control study investigated the microbiome in male AGA patients (*n* = 12) with stage III–IV alopecia per the Norwood–Hamilton classification [22]. Compared to healthy controls, diseased skin showed a significant increase in *C. acnes* (84% to 79%) and *Stenotrophomanas geniculata* (1.6% vs. 0%), and a significant decrease in *Staphylococcus epidermidis* (10% vs. 12%). Although alpha diversity did not differ, the ratio of *C. acnes* to *S. epidermidis* was significantly higher in patients with AGA.

#### 3.1.4. Diaper Dermatitis

Diaper dermatitis (DD), otherwise known as diaper rash, is an acute inflammatory reaction, usually secondary to diaper use. DD occurs in between 7 and 50% of the general population, and mostly in infants and elderly adults affected by urinary incontinence or of sedentary status [45]. A recently recognized component of DD pathogenesis is skin microbiome composition, with pathogenic strains such as *Candida* albicans and *S. aureus* being most commonly recognized with the inflammatory response [46].

One 2019 study demonstrated that compared to healthy infants and toddlers, those with DD had a significantly increased Shannon alpha diversity and Choas index, suggesting that increased skin microbiome diversity may play a role in DD [23]. Infants and toddlers with DD had significantly increased abundances of *Proteobacteria*, *Enterococcus*, *Erwinia*, *Pseudomonas*, *Rhodococcus*, *Acinetobacter*, and *Ruminococcus* and decreased abundances of *Clostridium* and *Actinomyces* compared to healthy controls. Furthermore, PCoA distribution in healthy samples were more concentrated than in DD samples, suggesting higher intra-group similarities in healthy skin [23]. Overall, more research into the role of the skin microbiome in DD is needed.

#### 3.1.5. Hand Eczema

Hand dermatitis, also known as hand eczema, is a common, multifactorial disease that is prevalent in 2 to 10% of the general population [47]. Evidence for the role of the skin microbiome in hand dermatitis is still emerging. However, one 2021 study evaluated the differences in skin microbiome profiles between health-care workers with hand eczema and healthy controls [24]. In both parts of the study, there was no difference found in alpha or beta diversity between the two cohorts. There were also no significant differences in bacterial species or genera [24]. A 2022 study demonstrated that the skin microbiome in hand eczema had a lower bacterial alpha diversity compared to healthy skin (*p* = 0.003) [25]. This study also found that the relative abundance of *S. aureus* in those with hand eczema was significantly higher than healthy skin (*p* < 0.001), and that disease severity was correlated with the abundance of *S. aureus* [25].

#### 3.1.6. Lamellar Ichthyosis

Lamellar Ichthyosis (LI) is a potential life-threatening skin disorder characterized by scaling, hyperkeratosis, and inflammation [48,49]. Current research on LI shows an increase in *C. acnes*, *Staphylococcus*, *Corynebacterium*, and *Malassezia* [50]. The pathogenesis and profiling of LI has been found to have a T helper 17 cell immune polarization [51,52]. Microbiome studies have indicated that *Staphylococci*, *Corynebacteria*, *M. slooffiae*, and *Trichophyton* may promote the T helper 17 cell skewing, while *C. acnes*, *M. globosa*, and *M. sympodialis* may support a homeostatic host–microbe interaction [50].

One comparative retrospective study observed the microbiome of patients with LI [26]. The microbiome of diseased skin compared to healthy controls showed an increased amount of methicillin-resistant *S. aureus* (MRSA), Fusobacterium (16.67% versus 4.17%), Gram-negative rods consisting of *Enterobacter*, *Proteus*, and *Klebsiella* (52.78% versus 51.39%), and fungal population mostly highlighting *Candida* (22.22% versus 5.56%). On the other hand, there was a decrease in lipophilic diphtheroids (11.11% versus 27.78%), *P. acnes* (5.6% versus 15.28%), and *Micrococci* (22.22% versus 36.11%), comparing composition percentages of patients with LI to healthy controls, respectively. MRSA was exclusively seen in patients affected with LI, constituting 33.33% of *S. aureus* flora.

#### 3.1.7. Psoriasis

Psoriasis is a chronic, inflammatory skin condition that affects approximately 3% of adults in the United States [53]. Psoriasis is classically characterized by symmetrically distributed well-circumscribed, erythematous scaly plaques involving the extensor surfaces, trunk, and scalp [54]. There is emerging evidence supporting the role of the skin microbiome and mycobiome in disease pathogenesis. For example, studies have demonstrated decreases in microbiome diversity, and altered skin microbiome profiles in psoriasis skin [55].

To the best of our knowledge, there is one study from 2015 that demonstrates shifts in the skin microbiome in psoriatic skin compared to nonlesional or healthy skin. In the 2015 study, psoriatic skin was found to have an increased abundance of *Firmicutes* phylum and decreased abundance of Proteobacteria phylum compared to healthy controls [27]. Moreover, there were no significant differences noted in Shannon alpha diversity or richness when comparing lesional versus nonlesional skin [27]. More research is needed to identify further shifts in psoriatic skin microbiomes and how these differences may affect therapeutic modalities for psoriasis.

#### 3.1.8. Rosacea

Rosacea is a chronic inflammatory skin condition that commonly affects the nose, chin, cheeks, and forehead characterized with flushing, erythema, telangiectasia, papules, and pustules [56]. Microorganisms on the skin can activate the innate immune system through the production of Toll-like receptor 2 [57]. This receptor can further elicit inflammation, erythema, and telangiectasia [58], which commonly present in rosacea. The pathogenesis of rosacea has also been associated with a decrease in the abundance of *Cutibacterium acnes* [59]. *C. acnes* has a protective effect by breaking down the sebum into free fatty acids, which can inhibit biofilm formation by bacteria such as *S. epidermidis* [60]. This prevents other harmful microorganisms from colonizing the skin.

Two observational case–control studies observed the microbiome shift in patients affected with rosacea and compared it to healthy controls. Both studies found no significant difference in skin microbiome diversity, in which one study looked at Shannon diversity, Chao, and Simpson index. In this study, there was an increased abundance of *S. epidermidis* (19.64% vs. 6.48%) in diseased skin, and a decreased actinobacteria (69.07% vs. 86.09%), *C. acnes* (61.79% vs. 79.69%), and firmicutes (8.05% vs. 21.19%) in diseased skin [28]. The other case–control study also found a decrease in *C. acnes*, but it was only observed in male patients [29]. There was an increase in the relative abundance of *C. acnes* in female patients. This study looked at alpha and beta diversities and discovered that across all ages, *C. acnes* remained the most abundant species and Corynebacterium kroppenstedtii was the second most abundant.

#### 3.1.9. Seborrheic Dermatitis

Seborrheic dermatitis (SD) is an inflammatory skin condition that presents with papulosquamous morphology in sebum-rich areas [61]. The pathophysiology of SD highly involves an impaired immune reaction to *Malassezia* species, which trigger inflammation and hyperproliferation of the epidermis. Specifically, *Malassezia* degrades the sebum to disrupt the lipid balance of the skin surface [62]. Recent research has linked SD disease states with an increase in *Malassezia* and Staphylococcus abundance [63,64].

One prospective cohort study looked at the microbiome homeostasis of both bacteria and fungi in seborrheic dermatitis through 16S rRNA sequencing and linear discriminant analysis effect size (LefSe), respectively [30]. Shifts in the microbiome showed an increased amount of five fungal genera (*Malassezia*, *Alternaria*, *Nagnishia*, *Hanseniaspora*, *Cladophialophora*) and five bacterial genera (*Staphylococcus*, *Blautia*, *Bifidobacterium Xylanimicrobium*, *Fusobacterium*, *Lysobacter*). On the contrary, there was lower enrichment of four fungal genera (*Mycosphaerella*, *Cladosporium*, *Rhodotorula*, *Debaryomyces*). Finally, there was a significant decrease in Shannon diversity, PD_Whole_tree index, and relative abundance of microorganisms (Table 2).

## 4. Discussion

This scoping review highlights several key findings regarding the role of skin microbiome in dermatological diseases. Across nearly all skin conditions examined, Staphylococcus aurerus was consistently found to be elevated in diseased skin, suggesting a common microbial signature associated with skin pathology. Most studies, except those focused on Acne vulgaris, also reported decreased microbial diversity in affected skin areas compared to healthy controls. These findings suggest that both microbial imbalance and dominance of pathogenic species, particularly S. aureus, may contribute to disease progression.

Given recent advances in biologics and small molecule therapies targeting the immune mechanisms of skin diseases such as AD and psoriasis [65,66], our review underscores the parallel importance of addressing microbial dysbiosis as part of a comprehensive treatment approach. For example, *S. aureus* in AD has been shown to secrete virulence factors such as toxins and proteases, which can further aggravate the skin barrier and disrupt the existing immune response [67]. Its frequent dominance in AD often reflects colonization rather than active infection, with pathogenicity varying by strain. CC1, the most common lineage in AD, carries multiple virulence factors (e.g., SEB, SEC, PVL) and shows enhanced fibrinogen binding, potentially contributing to greater disease severity. In contrast, CC30, more often found in healthy skin, produces fewer toxins and demonstrates lower virulence potential. These strain-specific differences underscore the importance of distinguishing colonization from infection when interpreting the role of *S. aureus* in AD.

Our scoping review identified *S. aureus* overrepresentation across all skin conditions examined, suggesting that reducing its abundance may be a therapeutic target. Notably, recent studies indicate that other Staphylococci strains can help inhibit *S. aureus* growth and pathogenicity by blocking quorum sensing—a cell–cell communication process that allows both Gram-negative and Gram-positive bacteria to regulate their gene expression [68,69]. With most studies, except those investigating acne vulgaris, showing a decreased microbiome diversity in diseased skin, restoring a balanced skin microbiome may help limit *S. aureus* burden and support skin health. However, caution is warranted in attributing a causative role to *S. aureus*. The microbiome differences observed between diseased and healthy skin may be a consequence of underlying pathology rather than its initiating factor. This distinction is critical when considering therapeutic strategies aimed at reducing *S. aureus* abundance or restoring microbial diversity. While the evidence suggests the potential of commensal-mediated quorum sensing inhibition to curb *S. aureus* pathogenicity, it remains unclear whether such modulation can alter disease trajectory. Addressing this uncertainty will require well-designed longitudinal and interventional studies to determine whether shifts in microbial composition actively drive disease progression and to evaluate the efficacy of targeted interventions, including probiotics, in improving patient outcomes.

The topic of altering the skin microbiome naturally leads to the discussion of oral and topical antibiotics. Oral probiotics, on the other hand, have shown promising results to become a viable therapeutic option for treating acne vulgaris while reducing the number of adverse events associated with prolonged use of antibiotics [70]. Such adverse events include drug fever, rash, lipodystrophy, nausea, vomiting, Stevens–Johnson syndrome, ototoxicity, and hypersensitivity reactions [71]. Finally, the use of antibiotics brings upon an urgent public health issue—antibiotic resistance [72]. The chronic usage of antibiotics has led to the development of drug-resistant diseases which claim over 700,000 lives annually [73]. The need to utilize non-antibiotic treatment has never been greater. Given the more recent research results and a stronger understanding in the increases and decreases in specific bacterial and fungal strains associated with respective skin diseases, probiotics hold potential in alleviating and restoring the microbiome of diseased skin. Although both topical and oral probiotics can help restore the skin microbiome, challenges in utilizing topical probiotics include environmental conditions that can prevent colonization of the probiotic [74].

Current biotic treatments that modulate the skin microbiome include topical and oral probiotics. Probiotics are live, nonpathogenic microorganisms that can help improve the skin microbiome balance and homeostasis [75]. Although additional research is needed to confirm the efficacy of probiotics, both topical and oral probiotics have been found to be effective in treating inflammatory skin diseases and may potentially improve wound healing and treatment of skin cancer [76]. An important theory of the alteration of skin microbiome through biotic treatment is the gut–skin axis, a co-dependent relationship between the gut microbiome and skin health. Dysbiosis of both the skin and the gut presents an immune imbalance, which can further progress the development of inflammatory skin diseases [77]. In addition, metabolites produced by the gut microbiome can disrupt the skin microbiome through the accumulation of bacterial toxins, antigens, and pathogens that can penetrate the epidermal barrier through blood circulation [78]. Both animal and human studies have demonstrated that probiotics could help improve inflammatory skin conditions such as psoriasis through the suppression of pro-inflammatory cytokines [79,80]. Clinical trials on the development of AD in infants found that probiotic supplementation can help reduce the risk of developing AD and respiratory allergic diseases in the future [81,82]. Probiotics are viable treatments and should be used as a safer and more financially reasonable alternative to antibiotics. However, variability in formulations, dosing, and study design limits broad generalization of these results. Hence, the concept that probiotics serve as a safer, cost-effective alternative to antibiotics remain a hypothesis requiring further validation. Nevertheless, the accumulating body of preliminary evidence, coupled with the expanding toolkit of microbiome-based therapeutics, provides a strong foundation for future clinical research to more precisely define the role of probiotics and related interventions in dermatologic care.

The studies reviewed here predominantly utilized 16S and ITS sequencing methodologies. There are several methodologies that can be utilized for microbiome-based sequencing such as 16S, ITS, short vs. long chain reads, and shotgun whole-genome sequencing, each with its benefits and limitations. 16S sequencing can identify bacteria at the genus and species level but typically cannot resolve to the strain level, and it cannot detect fungi or yeast. ITS sequencing, in contrast, can identify fungi and yeast but not bacteria [1]. Short-read 16S sequencing is faster but offers lower taxonomic resolution, whereas long-read 16S sequencing can achieve strain-level resolution but still cannot assess fungi or yeast and typically requires more time and resources. Shotgun whole-genome sequencing provides the most comprehensive profile, enabling strain-level identification across bacteria, fungi, and yeast, but it is more costly and computationally demanding [2].

## 5. Future Studies

Future work should extend beyond live probiotics to systematically investigate postbiotic and synbiotic approaches. Postbiotics—non-viable microbial products such as short-chain fatty acids, bacteriocins, and extracellular vesicles—offer advantages including improved stability, easier storage, and reduced infection risk [75,76,77,78]. Synbiotics may provide additive benefits by combining probiotics with prebiotics that support their activity.

Innovative delivery strategies also warrant exploration. Jiang et al. (2024) demonstrated that low-frequency ultrasound (LFS) enhances dermal penetration and directly modulates keloid fibroblast biology via Piezo1 activation, influencing calcium influx, migration, collagen production, and apoptosis [83]. Pairing LFS with microbiome-derived metabolites could both improve cutaneous delivery and engage fibroblast signaling pathways in fibrotic skin disease. Given the historical difficulty in distinguishing white patchy skin lesions, the utilization of deep convolutional neural networks may assist researchers in improving their analysis of various dermatological conditions especially when comparing lesions with or without the transformer modules applied [84]. To further fine-tune the microbiome research, a Cross-Modal Causal Representation Learning framework can be supplemented to reduce bias from microbiome feature sets [85].

Another priority is microbiome-informed risk prediction for immune-related adverse events. In pembrolizumab-induced psoriasis, Th17/IL-23-driven inflammation was identified, but microbial contributions remain unexamined [86]. Prospective immune checkpoint inhibitor cohorts with longitudinal gut and skin microbiome profiling, metabolomics, and immune phenotyping could identify predictive microbial signatures, enabling targeted preventive interventions with postbiotics or synbiotics.

Finally, emerging spatial and single-cell technologies—including spatial transcriptomics, single-cell RNA sequencing, and spatial metagenomics—can map microbe–host interactions in situ [87]. Applying these to lesional and perilesional skin would clarify how microbial shifts shape local immune and stromal environments, accelerating the shift from generalized supplementation to precise, mechanism-guided, and personalized microbiome-based dermatologic therapies.

## 6. Limitations

It is important to note the variability in each study’s design and methods. While some studies utilized a randomization process, many were non-randomized or involved either a comparative retrospective study or prospective pilot study. In addition, most studies had an intervention with a treatment. Since we were only interested in the microbiome shift due to the dermatological disease itself, we looked solely at the pre-treatment microbiome diversity and density. Therefore, a lot of data was not provided regarding statistical analysis and comparisons of the pre-treatment microbiome. To note specifically in Section 3, some studies only have observational increases or decreases in compositions of the microbiome. Most of the studies decided to utilize 16S rRNA sequencing, which is a limitation because 16S rRNA sequencing only captures bacteria, whereas the skin microbiome consists of not only bacteria, but also bacteriophages, fungi, and archaea. One study did not have access to ample resources and therefore utilized qualitative bacterial cultures and sensitivity from swabs instead of 16S rRNA sequencing. Finally, there is a significant limitation in the characterization of healthy, lesional, and nonlesional skin. There should be a difference in the microbiome of nonlesional versus healthy skin. Some studies did not have a healthy control and instead looked at lesional versus nonlesional areas for comparisons in the microbiome composition and density.

## 7. Conclusions

In conclusion, the skin microbiome is significantly altered in the progression of numerous dermatological diseases. Diversity of the skin microbiome is decreased, and there is a greater shift in the proportion of pro-inflammatory bacterial and fungal strains. The microbiome offers valuable insight into disease mechanisms and may guide targeted interventions for inflammatory skin disorders. While preliminary evidence supports probiotic use for microbiome restoration, further research is essential to confirm efficacy, optimize formulations, and define clinical indications. Advancing this field could ultimately improve patient outcomes across a broad spectrum of dermatologic conditions.

## Figures and Tables

**Table 1:** Summary table of the studies included in the scoping review.

Author	Disease	Female to Male Ratio (F to M)	Mean Age (Years) ± SD	Study Design	Comparison Groups	Sample Size, *n*	Method of Sequencing	Increased	Decreased	Other Changes
Dreno [12]	Acne	15 to 11	23 ± 6.5	DB, split-face RCT	Lesional vs. nonlesional	26	16S rRNA sequencing	Increased *Staphylococcus* (33.87% vs. 26.85%) and *Firmicutes* (52.01% vs. 47.01%) in lesional skin	Decreased *Proteobacteria* (34.10% vs. 28.90%) in lesional skin	Similar Shannon alpha diversity index score
Coughlin [13]	Acne Vulgaris	12 to 4	NA	Prospective Pilot Study	Healthy vs. diseased	16	16S rRNA sequencing	Increased *Staphylococcus* and *Propionibacterium* in diseased skin	N/A	Alpha diversity was higher in diseased skin four sites (midline forehead, dorsum of the nose, medial left cheek, and chin)
Callewaert [14]	AD	24 to 29	NA	DB RCT	Lesional vs. nonlesional	53	DNA extraction, 16S rRNA V4 amplicon sequencing via Quantitative PCR	*Staphylococcus aureus* in lesional skin		Lower Shannon alpha diversity index score in lesional skin
Khadka [15]	AD	21 to 21	11	RCT	Healthy vs. diseased	42	16S rRNA sequencing	Increased *S. aureus*	Decreased Shannon alpha diversity	Relative abundance of *S. aureus* positively correlated with disease severity as measured by SCORAD (rho = 0.545) *S. epidermidis* and *S. hominis* were inversely correlated with SCORAD
Lee [16]	AD	NA	28.3 for healthy, 34.2 for severe AD	RCT	Healthy vs. diseased	20	16S rRNA sequencing	NA	Decreased *Cutibacterium* and *Lactobacillus* in diseased skin	Increased Human beta defensin 2 (hBD-2) and lower Shannon diversity index score in lesional skin
Chandra [17]	AD	32 to 17	10.5	Non-RCT	Lesional vs. nonlesional	49	16S rRNA sequencing, ITS1 RNA gene showed fungal composition analysis	Increased *Alternaria*, *Coniosporium*, *Debaryomyce*, *Capnodiales*	NA	Gram-positive *Corynebacterium kroppenstedtiian* and *Staphlycoccus pettenkoferi* showed significantly positive correlations with pathogenic *Candida* species in lesional skin; *Pseudomonas* spp. correlated significantly with pathogenic *Aspergillus* and *Candida* spp.
Gonzalez [18])	AD	NA	NA	Single-blind RCT	Healthy vs. diseased	35	16S rRNA sequencing	Increased *Staphylococcus aureus* and *Staphylococcus* species in lesional skin. Nonlesional generally had less than 25% composition of *Staphylococci* whereas lesional skin had 60–70%.	Decreased *Corynebacterium* and *Propionibacterium* in diseased skin	Increased baseline total bacteria density by approximately 10-fold, and decreased community richness and Shannon diversity index in diseased skin
Kwon [19]	AD	NA	NA	RCT	Lesional vs. nonlesional	18	16S rRNA sequencing	Increased *Staphylococcus aureus*, *Staphylococcus* species in lesional skin	Decreased Shannon Diversity in lesional skin	*Haemophilus parainfluenzae*, *Streptococcus pseudopneumoniae*, *P. acnes*, and *Corynebacterium pseudogenitalium* showed significant negative correlations with *S. aureus* in lesional skin
Krzysiek [20]	AD	NA	Median, 6.8 for AD group, 8.7 for healthy	Non-RCT	Healthy vs. diseased	60	CHROMagar plates for *S. aureus* and *Malassezia*	Increased *Staphylococcus aureus*, *Staphylococcus* species in AD skin; Increased *Malassezia* species in AD skin	Decreased *Corynebacterium urealyticum* in AD skin	The number of *S. aureus* on lesional skin positively correlated with severity of disease according to validated scoring systems
Zeng [21]	AD	4 to 8	17.08 ± 6.72	Split side RCT	Lesional vs. nonlesional	12	16S rRNA sequencing	NA	NA	Lower Shannon alpha diversity index score and negative correlation between SCORAD and Shannon diversity index score in lesional skin
Filaire [22]	Androgenetic Alopecia	0 to 24	50.5 ± 3.2 for AGA, 48.6 ± 2.1 for healthy	Non-RCT	Healthy vs. diseased	24	16S rRNA sequencing, ITS1 rRNA sequencing	Increased *Cutibacterium acnes* (84% vs. 79%) and *Stenotrophomanas geniculata* (1.6% vs. 0%) in diseased skin	Decreased *Staphylococcus epidermidis* (10% vs. 12%) in diseased skin	Alpha diversity did not differ and ratio of *Cutibacterium acnes* to *Staphylococcus epidermidis* was significantly higher in diseased skin
Zheng [23]	Diaper Dermatitis	NA	NA	Non-RCT	Healthy vs. diseased	85	16S rRNA sequencing	Significantly increased Shannon diversity and Chao index (richness) Significantly increased *Proteobacteria, Enterococcus, Erwinia, Pseudomonas, Rhodococcus, Acinetobacter*, and *Ruminococcus*	Significantly decreased *Clostridium* and *Actinomyces*	PCoA distribution in healthy samples were found to be more concentrated, indicating higher intra-group similarities
Kuwatsuka [24]	Hand Eczema	1 to 0	34.3	Non-RCT	Healthy vs. diseased	16	16S rRNA sequencing	NA	NA	No difference in alpha or beta diversity between hand eczema and control groups
Norreslet [25]	Hand Eczema	28 to 22	40.1 ± 11.7	Non-RCT	Healthy vs. diseased	50	16S rRNA sequencing	Significantly increased *S. aureus* in diseased skin versus healthy controls	Decreased bacterial alpha diversity in diseased skin	Disease severity was correlated with abundance of *S. aureus*
Singh [26]	Lamellar Ichthyosis	9 to 18	35.56 weeks	Comparative retrospective study	Healthy vs. diseased	27	Qualitative bacterial culture and sensitivities from swabs	Increased methicillin resistant *Staphylococcus aureus* (MRSA), *Fusobacterium* (16.67% vs. 4.17%), Gram-negative rods consisting of *Enterobacter, Proteus, and Klebsiella* (52.78% vs. 51.39%), and fungal population mostly involving *Candida* (22.22% vs. 5.56%) in diseased skin	Decreased lipophilic diphtheroids (11.11% vs. 27.78%), *Propionibacterium acnes* (5.6% vs. 15.28%), and *Micrococci* (22.22% vs. 36.11%) in diseased skin	MRSA exclusively seen in LI patients constituting 33.33% of *Staphylococcus aureus* flora
Martin [27]	Psoriasis	22 to 32	59 ± 13	Non-RCT	Lesional vs. nonlesional	54	16S rRNA sequencing	Increased *Firmicute* phylum compared to healthy controls	Decreased *Proteobacteria* phylum compared to healthy controls	No significant differences in Shannon diversity or richness between lesional and nonlesional skin
Xiong [28]	Rosacea	26 to 18	Median, 27 for rosacea, 26 for healthy	Observational case–control	Healthy vs. diseased	44	16S rRNA sequencing	Increased *Staphylococcus epidermidis* (19.64% vs. 6.48%) in diseased skin	Decreased *actinobacteria* (69.07% vs. 86.09%), *Cutibacterium acnes* (61.79% vs. 79.69%), and *firmicutes* (8.05% vs. 21.19%) in diseased skin	No significant difference in diversity, statistically insignificant differences in Shannon diversity, Chao, and Simpson index
Rainer [29]	Rosacea	28 to 10	NA, range 23–65	Observational case–control	Healthy vs. diseased	38	16S rRNA sequencing	Increased relative abundance of *Cutibacterium acnes* in diseased skin of female patients (29.7% vs. 27.8%)	Decreased relative abundance of *Cutibacterium acnes* in diseased skin of male patients (23.8% vs. 57.5%)	Across all age groups, *Cutibacterium acnes* remained the most abundant species and *Corynebacterium kroppenstedtii* the second. No significant differences in ecologic diversity of microbiota
Yu [30]	SD	NA	NA	Prospective Cohort	Healthy vs. diseased	92	16S rRNA sequencing, LEfSe analysis	Increased amount of 5 fungal genera (*Malassezia, Alternaria, Nagnishia, Hanseniaspora, Cladophialophora*) and 5 bacterial genera (*Staphylococcus, Blautia, Bifidobacterium Xylanimicrobium, Fusobacterium, Lysobacter*) in diseased skin	Decreased enrichment in 4 fungal genera (*Mycosphaerella*, *Cladosporium*, *Rhodotorula*, *Debaryomyces*) in diseased skin	Decreased Shannon diversity, PD_whole_tree index, and relative abundance of microorganisms in diseased skin

**Table 2:** Summary table of all dermatological conditions.

Disease	Increased	Decreased	Other Changes
Acne	Increased *Staphylococcus, Firmicutes, and Cutibacterium*	Decreased *Proteobacteria*	Inconclusive Shannon alpha diversity score differences
Atopic Dermatitis	Increased *Staphylococcus aureus*, *Staphylococcus* species, *Alternaria*, *Coniosporium*, *Debaryomyce*, *Capnodiales*, and *Malassezia* species	Decreased *Cutibacterium*, *Lactobacillus*, *Corynebacterium*, and *Propionibacterium*	Decreased Shannon diversity index
Androgenetic Alopecia	Increased *Cutibacterium acnes* (84% vs. 79%) and *Stenotrophomanas geniculata* (1.6% vs. 0%)	Decreased *Staphylococcus epidermidis* (10% vs. 12%)	Alpha diversity did not differ and ratio of *Cutibacterium acnes* to *Staphylococcus epidermidis* was significantly higher in diseased skin
Diaper Dermatitis	Increased *Proteobacteria, Enterococcus, Erwinia, Pseudomonas, Rhodococcus, Acinetobacter*, and *Ruminococcus*	Significantly decreased *Clostridium* and *Actinomyces*	Significantly increased Shannon diversity and Chao index (richness). PCoA distribution in healthy samples were found to be more concentrated, indicating higher intra-group similarities.
Hand Eczema	Increased *S. aureus*	NA	Decreased bacterial alpha diversity in diseased skin. Disease severity was correlated with abundance of *S. aureus.* No difference in alpha or beta diversity between hand eczema and control groups.
Lamellar Ichthyosis	Increased methicillin resistant *Staphylococcus aureus* (MRSA), *Fusobacterium* (16.67% vs. 4.17%), Gram negative rods consisting of *Enterobacter, Proteus, and Klebsiella* (52.78% vs. 51.39%), and fungal population mostly involving *Candida* (22.22% vs. 5.56%)	Decreased lipophilic diphtheroids (11.11% vs. 27.78%), *Propionibacterium acnes* (5.6% vs. 15.28%), and *Micrococci* (22.22% vs. 36.11%)	MRSA exclusively seen in LI patients constituting 33.33% of *Staphylococcus aureus* flora
Psoriasis	Increased *Firmicute* phylum	Decreased *Proteobacteria* phylum	No significant differences in Shannon diversity or richness
Rosacea	Increased *Staphylococcus epidermidis*	Decreased *Cutibacterium acnes*	No significant difference in diversity, statistically insignificant differences in Shannon diversity, Chao, and Simpson index
Seborrheic Dermatitis	Increased amount of 5 fungal genera (*Malassezia, Alternaria, Nagnishia, Hanseniaspora, Cladophialophora*) and 5 bacterial genera (*Staphylococcus, Blautia, Bifidobacterium Xylanimicrobium, Fusobacterium, Lysobacter*)	Decreased enrichment in 4 fungal genera (*Mycosphaerella*, *Cladosporium*, *Rhodotorula*, *Debaryomyces*)	Decreased Shannon diversity, PD_whole_tree index, and relative abundance of microorganisms in diseased skin

## Data Availability

Data can be provided upon request.

## Supplementary Materials

The following supporting information can be downloaded at: https://www.mdpi.com/article/10.3390/jcm14176137/s1.

## Abbreviations

The following abbreviations are used in this manuscript:

rRNA	Ribosomal RNA
WGS	Whole-genome sequencing
PRISMA-ScR	Preferred Reporting Items for Scoping reviews and Meta-analysis Extension for Scoping Reviews
MRSA	Methicillin resistant staphylococcus aureus
LI	Lamellar Ichthyosis
RCT	Randomized-controlled trial
AD	Atopic dermatitis
AGA	Androgenetic alopecia
LefSe	Linear discriminant analysis effect size
SD	Seborrheic dermatitis
SCORAD	Shannon diversity index and Severity Scoring of Atopic Dermatitis
hBD-2	Human beta defensin 2
DD	Diaper dermatitis
